# Safety and efficacy of the BRAF inhibitor dabrafenib in relapsed or refractory hairy cell leukemia: a pilot phase-2 clinical trial

**DOI:** 10.1038/s41375-021-01210-8

**Published:** 2021-03-17

**Authors:** Enrico Tiacci, Luca De Carolis, Edoardo Simonetti, Mara Merluzzi, Antonio Bennati, Vincenzo Maria Perriello, Alessandra Pucciarini, Alessia Santi, Alessandra Venanzi, Valentina Pettirossi, Gianluca Schiavoni, Luisa Tasselli, Stefano Ascani, Stefano Volpetti, Brunangelo Falini

**Affiliations:** 1 Hematology, University and Hospital of Perugia, Perugia, Italy; 2 Anatomic Pathology, University of Perugia and Hospital of Terni, Perugia, Italy; 3 Clinica Ematologica, Azienda Sanitaria Universitaria Integrata, Udine, Italy

**Keywords:** Targeted therapies, Hairy cell leukaemia

## To the Editor:

Hairy-cell leukemia (HCL) is a rare indolent B-cell neoplasm that responds very well to chemotherapy with purine analogs (cladribine and pentostatin), but relapses are frequent (up to 58% in younger patients) [1, 2] and progressively less sensitive to these myelotoxic and immune-suppressive drugs. Our discovery that the *BRAF*-V600E mutation is the genetic lesion underlying HCL [3–5] and shaping the morphologic, molecular and anti-apoptotic features of leukemic cells [6], opened the way to BRAF inhibition as targeted therapeutic strategy in this disease [7, 8].

Thus, we and others assessed the safety and efficacy of a short course of vemurafenib (an oral BRAF inhibitor clinically effective in BRAF-V600E + melanoma [9]) delivered to 50 evaluable relapsed/refractory HCL patients enrolled in two phase-2 clinical trials [10]. Vemurafenib (given for a median of 16 or 18 weeks) produced 96–100% overall responses (ORs) and 35–42% complete responses (CRs), all with measurable residual disease (MRD) [10]. In our trial with longer follow-up, the median survival free from relapse of cytopenias in the 25 patients reaching an OR was 9 months after the end of treatment [10].

Our pre-clinical studies on patients’ HCL cells showed a promising anti-leukemic activity also for dabrafenib [6], another oral reversible ATP-competitive BRAF inhibitor approved in *BRAF*-V600E + metastatic melanoma [11]. Dabrafenib anti-leukemic activity was anecdotally reported also in single HCL patients, but response was not thoroughly documented [12] or disease burden was relatively limited [13] and did not clearly meet standard criteria for initiating treatment in HCL [14]. Therefore, we conducted a pilot phase-2 academic single-center clinical trial (EudraCT-2014-001379-29) to prospectively assess dabrafenib safety and efficacy in 10 relapsed/refractory BRAF-V600E + HCL patients, including 2 who had been treated with vemurafenib within our previous trial [10].

Enrollment of the 10 patients (completed in 9 months) followed a Simon minimax statistical design aiming at an OR rate ≥60% (versus <20% as null hypothesis) with *α* = 0.05 and *β* = 0.2. Key inclusion criteria (see also Supplementary Appendix) were: (i) disease refractory to, or relapsing ≤2 years after, the first course of purine analog, or relapsing whenever after a second or later course, or unsuitable to chemotherapy for patient comorbidites and/or old age; and (ii) disease requiring treatment for cytopenia(s) (neutrophils <1500/mm^3^, platelets <100,000/mm^3^ and/or hemoglobin <11 g/dl).

Dabrafenib was given orally at its standard dose of 150 mg twice daily for 8 weeks, followed by additional 4 weeks if no CR was obtained after 8 weeks. Response assessment, including splenic ultrasound and bone marrow (BM) evaluation, was performed every 4 weeks of dabrafenib dosing. Blood counts and chemistry were obtained weekly during the first 4 weeks, every 2 weeks thereafter during treatment, and every 3 months during follow-up. To monitor for dabrafenib toxicities, patients also underwent dermatologic examinations every 4 weeks and electrocardiograms every 2 weeks during treatment.

As in our previous vemurafenib trial [10], CR required resolution of cytopenias (as defined above), no palpable splenomegaly and no leukemic hairy cells morphologically visible in the BM biopsy and peripheral blood smear. Partial remission (PR) required cytopenias resolution and ≥50% reduction of splenomegaly and HCL infiltration in the BM biopsy by immunohistochemistry. Minor response (MR) required an improvement of ≥50% in all abnormally low blood counts. Progression was defined as a HCL-related death, relapse or worsening of cytopenias after starting treatment, whichever occurred first.

The 10 patients (all males; median age: 62 years) had a median of 3.5 prior therapies, including a purine analog in all cases except one old patient previously treated with interferon (Table 1). Other previous treatments were interferon (6/10 patients, 60%), rituximab (3/10, 30%) as monotherapy (*n* = 2) or with pentostatin (*n* = 1), vemurafenib (2/10, 20%), and splenectomy (2/10, 20%). Median neutrophils were 455/mm^3^, platelets 56,000/mm^3^ and hemoglobin 11.1 g/dl. Median HCL infiltration in the BM biopsy was 80% (range 40–95%). Five of the 8 (63%) non-splenectomized patients had splenomegaly (longest spleen diameter: 14.5–28 cm).

**Table 1:** Demographic features, prior therapies and response to dabrafenib of the 10 HCL patients.

Pt.	Sex	Age	Previous therapies	Blood counts and spleen size:				Response to dabrafenib	Weeks of treatment until best response	Total weeks of treatment	Progression-free survival (in months^b^)	Treatment-free survival (in months^c^)
				Neut /mm^3^	PLT ×10^3^/mm^3^	Hb g/dL	Spleen^a^ cm					
1	M	55	IFN, CDA, CDA, IFN, RTX, RTX, IFN, DCF+RTX, Splenectomy	71	13	7.5	–	PR	8	12	42	56
2	M	52	IFN, CDA, CDA	959	55	13.8	16	PR	4	12	5.5	10
3	M	52	DCF, CDA	701	65	12.1	23.5	PR	12	12	7	13
4	M	63	CDA, CDA	231	56	14.2	11	PR	8	12	10	12
5	M	55	IFN, CDA, CDA, CDA	630	103	12.2	14.5	CR	12	12	15.5	31
6	M	79	Splenectomy, IFN, CDA, CDA, IFN, CDA, RTX, RTX, VEM, VEM	290	36	9.5	–	PR	8	12	7	9
7	M	75	CDA^d^	789	81	10.6	11	CR	8	8	60.5+	59+
8	M	61	CDA, RTX, IFN, DCF	80	11	8.6	28	MR	8	12	3.5+	0.27
9	M	81	DCF, DCF, CDA, VEM, VEM	500	95	10.8	13	CR	12	12	14	21
10	M	75	IFN	410	31	11.4	20	MR	4	12	7	14.5

*Neut.* neutrophils, *PLT* platelets, *Hb* hemoglobin, *IFN* interferon, *CDA* cladribine, *DCF* pentostatin (deoxycoformycin), *RTX* rituximab, *VEM* vemurafenib, *PR* partial response, *CR* complete response, *MR* minor response.

^a^Longest diameter.

^b^From the start of treatment.

^c^From the end of treatment.

^d^Primary refractory.

All patients received dabrafenib for 12 weeks, except one reaching CR after 8 weeks. The OR rate was 80%, including 3 CR (30%) and 5 PR (50%). Median time to recovery of platelets (≥100,000/mm^3^), neutrophils (≥1500/mm^3^) and hemoglobin (≥11 g/dl) in responding patients was respectively 15 days (range: 6–36), 35 days (range: 11–58) and 51 days (range: 43–64).

The clinical benefit of dabrafenib lasted relatively long in the 3 complete responders, despite all had MRD (~5–10% HCL cells by immunohistochemistry in the BM biopsy post-therapy). One CR patient is progression-free at 60.5 months. The other two patients formally progressed at 15.5 and 14 months from starting treatment, but required further therapy only later, respectively 31 and 21 months after ending dabrafenib. Indeed, as with vemurafenib [10], blood counts indicative of relapse per protocol (e.g., neutrophils <1500/mm^3^) can remain for a relatively long time above or around the thresholds commonly triggering anti-HCL therapy in routine care (e.g., neutrophils <1000/mm^3^) [14]. Furthermore, 1/3 CR patients had previously received a short vemurafenib treatment twice [10], which produced a CR in both instances but a shorter progression-free survival (PFS) with the second vemurafenib course (10.5 months) compared to the first one (16 months). Interestingly, PFS with dabrafenib was 14 months, comparing well with the last vemurafenib course.

Also 1/5 PR patients enjoyed a long survival free from progression (occurring at 42 months) and from a new therapy (started at 56 months), despite a previous splenectomy (which seems to associate with lower efficacy of vemurafenib [10] and moxetumomamb pasudotox [15]). Conversely, in 4/5 PR patients, progression occurred 5.5-10 months after starting treatment and was followed by another therapy in 3 cases (10-13 months after ending dabrafenib) or death in 1 case (pneumonia 9 months after ending dabrafenib). The latter patient had previously achieved only PRs after being treated twice with vemurafenib.

The remaining 2/10 patients, although reaching only MR, had significant clinical benefit from dabrafenib. In particular, one severely pancytopenic patient with huge splenomegaly (28 cm) and high transfusion requirement (4 units of erythrocytes and 4 of platelets per month), quickly became transfusion-independent (within 10 days from starting treatment) and did not eventually qualify for PR just because of residual mild thrombocytopenia (70,000/mm^3^). However, the beneficial effect of dabrafenib, including considerable reduction of splenomegaly (17.5 cm), allowed him to safely undergo a laparoscopic removal of the spleen, which would have been hardly feasible at baseline and which got rid of the main residual disease reservoir (as BM leukemic infiltration post-dabrafenib was relatively mild). Indeed, splenectomy resulted in resolution of thrombocytopenia and freedom from relapse at 56 months. Also the other minor responder (75-year old) did not qualify for PR because of mild thrombocytopenia (80,000/mm^3^), and enjoyed a relatively long treatment-free survival (14.5 months).

**Fig. 1:**
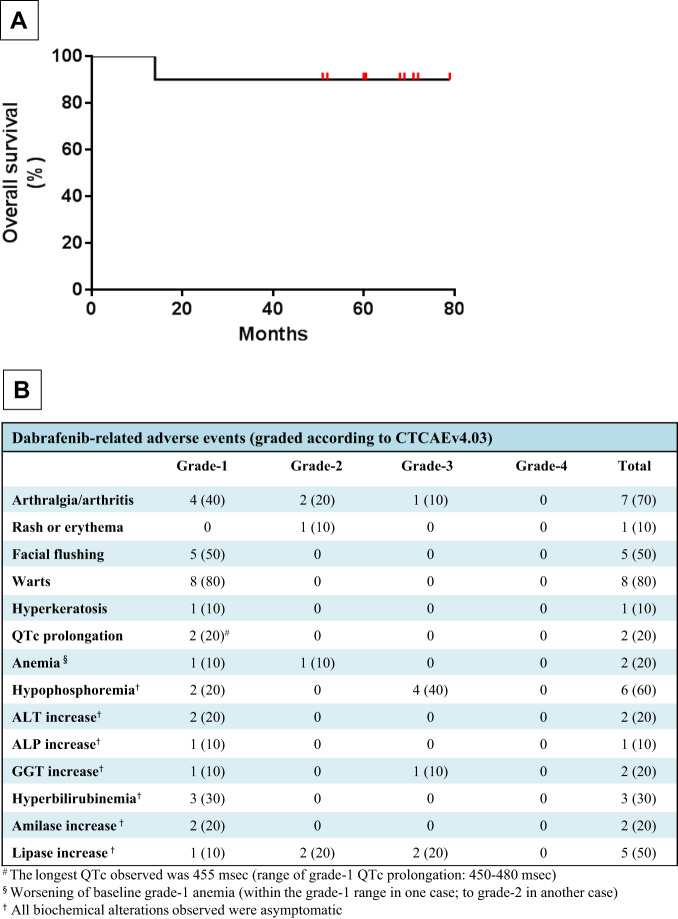
Survival and toxicity after dabrafenib treatment in relapsed/refracotry HCL. **A** Overall survival of the 10 patients treated with dabrafenib. **B** Toxicities of dabrafenib in the 10 patients.

Overall survival in the 10 patients was 90% at a median follow-up of 64 months from starting treatment (range: 14–79) (Fig. 1A).

Dabrafenib-related toxicities (Fig. 1B) were similar to those observed in melanoma trials [11] and were mostly of grade-1/2, none of grade-4 and none leading to permanent drug discontinuation. They were all reversible and usually represented by arthralgias, facial flushing, warts, and asymptomatic increase of the QTc interval or of liver/pancreatic function tests. As with vemurafenib [10], no significant myelosuppression was observed. Four patients (40%) received the full dabrafenib dose (150 mg b.i.d.) for the whole planned duration, whereas in 6 patients (60%) the dose was reduced (to 100 mg b.i.d., *n* = 5; to 50 mg b.i.d., *n* = 1) for a total of 29-56 days per patient, due to grade-2 arthralgia, grade-3 arthritis or asymptomatic laboratory abnormalities (grade-2/3 lipase increase; grade-3 phosphate decrease; grade-1 QTc prolongation). Most of these patients (4/6) were then able to re-escalate the dose to 150 mg b.i.d. Thus, the median dose intensity actually delivered to the 10 patients (relative to the intended dose of 150 mg b.i.d. for the planned duration of 8–12 weeks) was high at 88% (range: 78–100%).

The spectrum of dabrafenib toxicities in HCL patients was largely similar to vemurafenib. However, in an indirect comparison (and considering that vemurafenib was given for a median of 16–18 weeks [10], versus 12 weeks for dabrafenib), rash seemed to occur less frequently with dabrafenib (1/10 cases 10%; grade-2) than with vemurafenib (29/54 cases, 54%; grade-2, *n* = 22 cases; grade-3, *n* = 7 cases) (*p* value 0.015 by Fisher’s exact test). Furthermore, no cutaneous neoplasms (a known complication of prolonged treatment with BRAF inhibitors [16]) occurred in dabrafenib-treated patients, versus 7/54 (13%) patients treated with vemurafenib [10]. Moreover, only one dabrafenib-treated patient (10%) developed symptomatic grade-3 toxicity (elbow arthritis), compared to 17/54 (31%) patients with vemurafenib [10]. Finally, the drug dose had to be reduced for most (≥50%) of the planned treatment duration in 1/10 (10%) patients treated with dabrafenib (due to grade-2 arthralgia), compared to 9/27 (33%) evaluable HCL patients in our vemurafenib trial [10].

In conclusion, a short treatment with dabrafenib safely and quickly induced objective responses and significant clinical benefit in all relapsed/refractory HCL patients enrolled in this pilot study, including a few previously treated with vemurafenib. The anti-leukemic activity of dabrafenib appears overall similar to vemurafenib [10], with a possibly milder toxicity profile. Thus, dabrafenib may represent a valid alternative to vemurafenib both in HCL patients naive to a BRAF inhibitor and in those previously intolerant to vemurafenib (as shown in melanoma patients) [17]. CR depth with vemurafenib [10] or dabrafenib in relapsed/refractory HCL is lower (no MRD-negativity) compared to the anti-CD22 immunotoxin moxetumomab pasudotox (35% MRD-negative CR rate) [15], although we cannot exclude that BRAF inhibitor activity could deepen with more prolonged dosing. However, a short treatment combining vemurafenib with rituximab seems to produce superior results in relapsed/refractory HCL (61% MRD-negative CR rate [18]), and studies assessing the safety and efficacy of dabrafenib plus anti-CD20 immunotherapy are warranted.

## Supplementary information

Supplementary appendix
