# Supplementary material for: Safety and efficacy of the BRAF inhibitor dabrafenib in relapsed or refractory hairy cell leukemia: a pilot phase-2 clinical trial
Source: Leukemia. 2021 Mar 17;35(11):3314–8. doi: 10.1038/s41375-021-01210-8 (PMC8550943; doi:10.1038/s41375-021-01210-8)
Supplement: Supplementary file 1 — Supplementary appendix [file 41375_2021_1210_MOESM1_ESM.doc]

**Supplementary Appendix**

Main inclusion criteria in this trial were:

- Age ≥18 years
- ECOG-PS 0-2
- Adequate renal/hepatic function, i.e. creatinine ≤2 times the upper limit of normal (ULN;) AST, ALT and ALP ≤2.5 times the ULN; bilirubin ≤1.5 times the ULN
- Proven diagnosis of HCL harboring the BRAF-V600E mutation
- Disease refractory to, or relapsing within 2 years from, the first course of purine analog; or disease relapsing whenever after a second or later course; or disease unsuitable to chemotherapy for patient comorbidites and/or old age.
- Disease requiring treatment for cytopenia(s) (neutrophils <1,500/mm^3^, platelets <100,000/mm^3^ and/or hemoglobin <11 g/dl), opportunistic infections or bulky/symptomatic splenomegaly.

None of the patients enrolled had an active infection, although the latter was not an exclusion criterion.
